# Autoimmune-mediated hypertriglyceridemia in a young male with refractory immune thrombocytopenia: a case report

**DOI:** 10.1016/j.jlr.2026.101162

**Published:** 2026-09-30

**Authors:** Batya Green, Eran Leitersdorf, Shoshana Shpitzen, Rayan Alyan, Daniel Schurr, Aviv Talmon, Yuval Tal, Merielle Kaplan, Ram Weiss, Ronen Durst

**Affiliations:** 1 Hadassah Medical Center, Jerusalem, Israel; 2 Rambam Medical Center, Haifa, Israel

**Keywords:** APOC3, GPIHBP1 antibodies, pancreatitis, hypertriglyceridemia

## Abstract

Severe hypertriglyceridemia (HTG), defined as triglyceride levels >500 mg/dl can cause acute pancreatitis and other metabolic complications. While primary forms are typically genetic, autoimmune-mediated HTG is rare. We report a 24-year-old male with refractory immune thrombocytopenia who developed severe HTG due to anti-GPIHBP1 autoantibodies. Conventional therapies, including plasmapheresis and immunosuppressive agents, were insufficient. Compassionate use of plozasiran, an RNA interference agent targeting APOC3, achieved an 80% reduction in triglyceride levels. This case highlights the importance of recognizing autoimmune GPIHBP1-mediated hypertriglyceridemia, as identifying the underlying mechanism may enable targeted therapy.

Autoimmune hypertriglyceridemia due to anti-glycosylphosphatidylinositol anchored high density lipoprotein binding protein 1 (anti-GPIHBP1) antibodies is a rare, acquired disorder characterized by severe HTG. GPIHBP1 is an endothelial protein that shuttles lipoprotein lipase (LPL) to the capillary lumen, enabling hydrolysis of triglyceride-rich lipoproteins (TRL) (1). Autoantibodies that block this transport result in accumulation of very-low-density lipoprotein and chylomicrons, often leading to triglyceride levels exceeding 1,000 mg/dl and recurrent pancreatitis. This autoimmune phenomenon mimics genetic deficiency in LPL activity and carries similar risk for acute pancreatitis (2).

This autoimmune mechanism has been described in multiple case series and is associated with both primary and secondary autoimmune conditions, including systemic lupus erythematosus, immune thrombocytopenic purpura (ITP), and thyroid disease 2, 3, 4. Conventional triglyceride-lowering therapies-, such as statins, fibrates, and omega-3 fatty acids-have limited efficacy in this setting and have not demonstrated a reduction in pancreatitis risk in randomized control trials 2, 4.

## Materials and methods

A 24-year-old Muslim male with no family history of dyslipidemia presented in 2018 with severe ITP, manifesting as platelet counts below 6,000/μl, easy bruising, and bleeding gums. Laboratory evaluation revealed triglyceride levels of 4,500 mg/dl. Childhood lipid panels were normal, excluding congenital hypertriglyceridemia. As per guidelines, the patient received adequate treatment for his HTG including a high potency statin, fibrates and omega 3 fatty acids (5).

His ITP was refractory to multiple therapies, including corticosteroids, IV immunoglobulin, and rituximab, ultimately necessitating splenectomy. Hypertriglyceridemia persisted despite immunosuppressive therapy (rituximab, mycophenolate mofetil, azathioprine) and supportive measures including low-molecular-weight heparin. Weekly plasmapheresis was required to manage recurrent pancreatitis. Despite these efforts the patient experienced 8 episodes of life-threatening recurrent pancreatitis between 2018-2023, was in critical condition in the ICU and required many months of rehabilitation in between episodes. He also had endocarditis due to infected indwelling IV catheter used for plasma pheresis in May of 2025.

Genetic testing, including whole-exome sequencing, did not identify mutations associated with familial chylomicronemia syndrome (FCS), including in the *GPIHBP1* gene. Pathogenic variants in *GPIHBP1* are a recognized cause of familial hypertriglyceridemia 6, 7. In 2023, serum ELISA confirmed the presence of anti-GPIHBP1 antibodies, establishing an autoimmune etiology. The ELISA assay used to detect anti-GPIHBP1 autoantibodies had no established reference range and was intended for in vitro diagnostic use; therefore, the patient’s result was interpreted in comparison with that of a randomly selected control patient (2).

Compassionate use approval was obtained for plozasiran, an RNA interference therapy targeting *APOC3* 8, 9, in July of 2023. APOC3 is a glycoprotein produced primarily in the liver that is a key regulator of triglyceride clearance and metabolism through its inhibition of LPL.

Furthermore, ApoC-III impairs the hepatic clearance of TRLs through mechanisms that are independent of LPL activity. This is supported by the substantial reduction in triglyceride levels observed in patients with FCS treated with antisense oligonucleotides targeting *APOC3*, despite their severe or complete LPL deficiency. The suggested mechanism is that APOC-III displaced APOE from the surfaces of remnant particles, thereby preventing interaction with hepatic receptors (9).

After administration of the first injection, a 65% reduction in TG levels to 1,370 mg/dl was observed, and after receiving 5 doses of 25 mg subcutaneouly as 3 month intervals, he achieved an 80% reduction in triglyceride levels from reaching a new steady state, at 900 mg/dl as seen in Fig. 1. To date the patient has not experienced another episode of acute pancreatitis and no longer requires plasmapheresis. The treatment was well tolerated by our patient with no occurrence of adverse events.

**Fig. 1:**
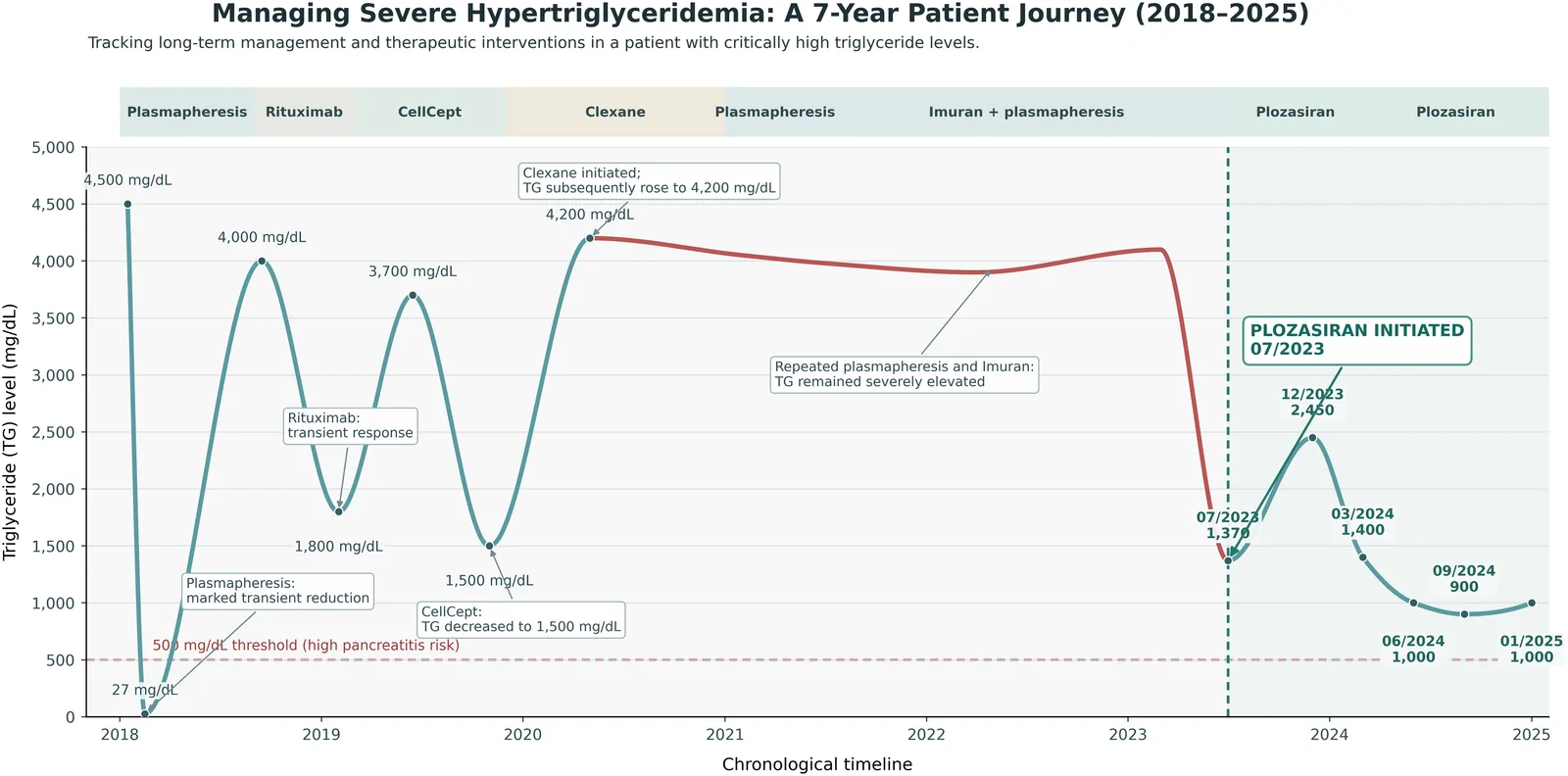
Managing severe hypertriglyceridemia: a 7-year patient journey (2018–2025).Tracking the long-term management and therapeutic interventions for a patient with critically high triglyceride levels. This timeline illustrates a complex case of severe hypertriglyceridemia over seven years, characterized by triglyceride levels often exceeding 500 mg/dL and significantly increasing pancreatitis risk. The journey explores initial acute crises managed with mechanical plasmapheresis, followed by various traditional immunosuppressants, and concludes with the introduction of the novel RNAi therapy, plozasiran, showing sustained stabilization. TPE, therapeutic plasma exchange.

## Discussion

We describe a case of autoimmune severe HTG, caused by autoantibodies against GPIHBP1, in the setting of ITP. Anti-GPIHBP1 antibodies disrupt LPL transport, causing severe triglyceride accumulation 1, 4. As seen in FCS, conventional lipid-lowering therapies are often insufficient to reduce risk for pancreatitis (7). Despite the autoimmune etiology, the patient’s ITP and HTG both were refractory to immunosuppression, as shown in previous case series (1).

Plozasiran inhibits *APOC3*, a protein that impairs triglyceride clearance both via LPL and LPL independent inhibition. 8, 9.

In patients with anti-GPIHBP1 antibodies, LPL transport is blocked, yet *APOC3* inhibition facilitates alternative clearance pathways for TLRS. This finding is most likely attributable to LPL-independent pathways mediating the clearance of TRLs, as has been demonstrated in patients with FCS. Clinical trials have demonstrated up to 80% reductions in triglyceride levels and a decreased risk of pancreatitis when *APOC3* is inhibited in patients with genetic or clinically diagnosed FCS 10, 11, 12).

Plozasiran, an emerging RNA interference therapy targeting *APOC3*, may provide substantial benefit even when the underlying mechanism differs from classical genetic forms. Early recognition is critical to prevent recurrent pancreatitis and guide targeted therapy.

## Conclusion

Autoimmune anti-GPIHBP1 antibodies are a rare cause of secondary HTG. Clinicians should consider this diagnosis in young patients with autoimmune disorders and severe HTG. RNA interference therapy targeting *APOC3*, such as plozasiran, was used in this case as a therapeutic strategy to mitigate the severe hypertriglyceridemia resulting from immune-mediated LPL dysfunction, by enhancing LPL-independent clearance of TRLs. —The use of plozasiran in this clinical setting remainsinvestigational,but may represent a promising therapeutic strategy when conventional approaches fail, underscoring the potential role of precision medicine in autoimmune dyslipidemia.

## Data availability

The data underlying this article cannot be shared publicly because they contain information that could compromise patient privacy. De-identified data may be made available from the corresponding author upon reasonable request and with appropriate ethical approval.

## Conflict of interest

The authors declare that they have no conflicts of interest with the contents of this article.
